# Modelling marsupial mastication: The biomechanical bite model of the Linnaeus's mouse opossum *Marmosa murina* (Marsupialia, Didelphidae)

**DOI:** 10.1111/joa.70003

**Published:** 2025-06-27

**Authors:** Vincent Decuypere, Anthony Herrel, Quentin Grimal, Damien Germain, Anne‐Claire Fabre, Sandrine Ladevèze

**Affiliations:** ^1^ Centre de Recherche en Paléontologie—Paris UMR 7207 CR2P Sorbonne Université Paris France; ^2^ Laboratoire d'Imagerie Biomédicale UMR 7371 LIB Sorbonne Université Paris France; ^3^ Mécanismes Adaptatifs & Evolution UMR 7179 MECADEV Muséum National d'Histoire Naturelle Paris France; ^4^ Department of Biology, Evolutionary Morphology of Vertebrates Ghent University Ghent Belgium; ^5^ Department of Biology University of Antwerp Wilrijk Belgium; ^6^ Naturhistorisches Museum Bern Bern Switzerland; ^7^ Institute of Ecology & Evolution Universität Bern Bern Switzerland

**Keywords:** anatomical description, Didelphidae, masticatory system, PCSA, static equilibrium

## Abstract

Marsupials have evolved alongside other mammals on many continents, mainly in the southern hemisphere, developing their own traits and adaptations. Although the relationships between morphology, bite force, and diet have been well studied in many vertebrate groups, this has rarely been the case for marsupials until recently. Present‐day American marsupials' diet and their feeding capacities, considered generalists, remain poorly understood. A better understanding of current American marsupials will lead to more accurate inference models for extinct metatherians. Here, we study and describe for the first time the masticatory apparatus of the Linnaeus' mouse opossum *Marmosa murina*, along with its performance. Bite forces data were collected for different marsupial species during a field mission in French Guiana in 2017. A 3D bite reconstruction model has been established through dissections and using the lever arm method, based on the static equilibrium of the muscular vectors in the jaw. The optimal gape angle and the contribution of each masticatory muscle to the closing of the mouth were determined. We identify and individualized the different fascicles of the masseter, zygomaticomandibular, temporal, and pterygoid muscles, together with their respective origin and insertion areas. The optimal gape is around 6°, supporting the use of the last molar to get the strongest bite forces. The *M. masseter superficialis*, the *M. temporalis superficialis*, and the *M. temporalis profundus medialis* are the muscles having the greatest impact on the maximum bite force. Our biomechanical model allows a correct approximation of the biting force. However, the muscle stress value has to be increased from 30 N.cm^−2^ to 44.360 N.cm^−2^ and 54.209 N.cm^−2^ to match the in vivo bite forces on the last molar (m4) for *Marmosa murina*. These high values are rather surprising, suggesting that our model, with the use of standardized constants for all mammals, underestimates true bite forces.

## Graphical abstract

The marsupial masticatory apparatus has rarely been studied until recently, mainly in Australasian species. We therefore reconstructed the maximum bite forces of the South American Linnaeus's mouse opossum *Marmosa murina* using *in vivo* bites, specimen dissections, and 3D static equilibrium of the jaw muscles. Our research provides relevant anatomical data and raises new evolutionary hypotheses and questions about the inner muscular or physiological parameters of the marsupial jaw.

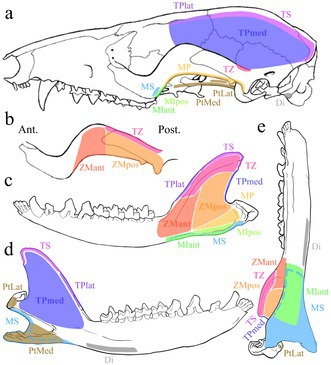

## 1 INTRODUCTION

The vertebrate skull shape is a complex and integrated system that is significantly influenced by a multitude of factors including the physical constraints related to locomotion and feeding, the protection of the brain and sensory organs (Dumont et al., 2016; Herrel et al., 2001; Meloro et al., 2015), as well as phylogenetic constraints (Dumont et al., 2016). In mammals, it has been shown that diet and feeding habits strongly impact the head system (e.g., Radinsky, 1981; Langenbach & Eijden, 2001; van Cakenberghe et al., 2002; Ross et al., 2007; Dumont et al., 2012; Law et al., 2018). Moreover, the ability to consume resistant food items is dependent on the generation of biting forces allowing a reduction of the food item before it can be swallowed (Berthaume, 2016). Species are therefore likely to have distinct cranial morphologies and adaptations depending on the degree of mechanical resistance of the food they typically consume. Indeed, in modern vertebrates, the relationship between diet, bite force, and morphology has been extensively studied in groups as diverse as lizards (Herrel et al., 1998a, 1998b), bats (Aguirre et al., 2003; Herrel et al., 2008; Santana et al., 2010), primates (Deutsch et al., 2020; Dickinson, Davis, et al., 2021; Dickinson, Pastor, et al., 2021; Perry et al., 2011), felids (Hartstone‐Rose et al., 2012), murids (Ginot et al., 2018), mustelids (Hartstone‐Rose et al., 2019), suids (Sicuro et al., 2021), and across the order Carnivora (Hartstone‐Rose et al., 2022). For all these groups, bite force is an excellent proxy of the diet of a species and can be recorded in vivo or estimated from the anatomy of the masticatory system.

To date, a handful of study recorded in vivo bite force (e.g., Druzinsky et al., 2016; Ginot et al., 2018; Hartstone‐Rose et al., 2019; Nogueira et al., 2009; Santana et al., 2010; Sicuro et al., 2021) and most of bite force were estimated using the anatomy of the masticatory system (e.g., Herrel et al., 2008; Van Daele et al., 2009). From an anatomical point of view, the size and configuration of the masticatory muscles should allow to determine the performance of the jaw system and maximal bite force. The number of muscle fibres, composed of serially arranged sarcomeres (Loeb & Ghez, 2000) in parallel, ultimately determines the force that a muscle can exert and is represented by the “physiological cross‐sectional area” (PCSA; Close, 1972). Species feeding on mechanically resistant material have been shown to have a higher average PCSA value than species feeding on softer materials (Hartstone‐Rose et al., 2012; Herrel et al., 2008; Perry et al., 2011). Moreover, the length of the muscle fibres of a fascicle determines the maximum potential gape that a species can achieve (Hartstone‐Rose et al., 2019) in addition to constraining mouth closing speed. Species consuming large preys can be expected to have longer fibres (Dumont & Herrel, 2003; Hartstone‐Rose et al., 2019). As these performances can be very informative about an animal's lifestyle and is critical to understand functional adaptations (O'Higgins et al., 2011), it is equally important to understand whether the bite force estimated from data on the morphology of museum specimens is comparable to the bite force in vivo and can therefore be used to more accurately reconstruct the paleobiology of extinct species.

In this prospect, marsupials represent an excellent model group, due to their varied behaviours and morphologies. Their evolutionary history extends back to the Jurassic period, and they have evolved in parallel with placentals, either through evolutionary convergences (e.g., Jones, 2003; Newton et al., 2021) or by obtaining their unique morphological adaptations, their gestation, development, and growth (e.g., Tyndale‐Biscoe, 2005). Their very wide mouth opening (75–80° for *Sarcophilus harrisii* (Boitard, 1841) and *Thylacinus cynocephalus* (Harris, 1808); see Paddle, 2000; Pemberton & Renouf, 1993) could be a plesiomorphic characteristic (Attard et al., 2011; 102–105.8° for the fossil metatherian *Thylacosmilus atrox* Riggs, 1933; see Churcher, 1985; Wroe et al., 2013). Although present‐day South American marsupials are considered generalists, *i.e*. feeding on fibrous plant material, fruits, vertebrates, and arthropods, some species show clear primary dietary preferences, such as piscivory for *Chironectes minimus* (Zimmermann, 1780) and carnivory for *Lutreolina crassicaudata* (Desmarest, 1804) (Astúa, 2015; Goin et al., 2015). Previous descriptions of the masticatory apparatus in marsupials have focused on Australian species (e.g., Crompton et al., 2008; Davison & Young, 1990; Thomas et al., 2024; Tomo et al., 2007; Wood Jones, 1949), while only a few studies have focused on American species (e.g., the dusky caenolestid, Osgood, 1921; the common opossum, Turnbull, 1970; the big lutrine opossum, Delupi et al., 1997; the white‐eared opossum, Abreu & Astúa, 2024). Furthermore, the behaviour and feeding habits of extant South American species remain poorly studied (Chemisquy et al., 2021; Tarquini et al., 2019). As such, a better understanding of the feeding behaviour and habits is importantly needed as it can shed the light on the lifestyle of these poorly known species that represent a major part of the marsupial diversity.

To better understand how estimated bite force from the anatomical data of the feeding system relate on in vivo bite force and can be informative about the feeding habit of a species, we studied the Linnaeus's mouse opossum *Marmosa murina* (Linnaeus, 1758). This small (average 38 g and 290 mm long), nocturnal, mainly arboreal marsupial mouse opossum with a long prehensile tail (average 170 mm long), lives in the low canopy of trees in Central and South America (Catzeflis et al., 2014). It is very commonly found in French Guyana, considered an insectivorous omnivore and as Least Concern (LC) on the IUCN Red List of Threatened Species (Brito et al., 2021). Although this species is easily accessible and handled, it has been poorly studied to date, given the difficulties of tracking this small marsupial in its environment (Costa et al., 2024). More specifically, we describe the muscular and skeletal anatomy of its masticatory system and compared it to other marsupials. On the basis of these data, a static bite model is established and compared with in vivo bite force measurements. As the reconstruction of bite forces in marsupials has rarely been conducted within mammals, the parameters of the biomechanical model are discussed, as well as the contribution of each masticatory muscle to the maximal bite force.

## 2 MATERIALS AND METHODS

### 2.1 Specimens

The morphology of the masticatory muscles of *Marmosa murina* was based on two individuals from the JAGUARS collection (“Joindre l'Amazonie et la Guyane: Animaux, Ressources et Sciences », Institut Pasteur of French Guiana”): M1496 and M2851, both adult male specimens. Bite force measurements were taken in the field in 2017 at (i) the incisors and (ii) the last molar ($F{\left(\text{alive}\right)}_{I}$ and $F{\left(\text{alive}\right)}_{M}$ respectively), using an isometric Kistler force transducer (type 9203, range ± 500 N; Kistler, Zurich, Switzerland) mounted on a purpose‐built holder and connected to a Kistler charge amplifier (Type 5995 A, Kistler; see Herrel et al., 1999).

### 2.2 Dissection and Anatomy

The specimens were fixed in 10% formalin, rinsed, and preserved in 70% ethanol. In addition to the jaw adductors, only the *M. digastricus*, which is part of the suprahyoid muscles and sometimes considered as a masticatory muscle, is observed and described here. For each muscle, the cranial origin and the mandibular insertion were carefully recorded. Photographs were taken throughout the dissection to document the jaw muscle anatomy. Each dissected muscle was isolated, labelled, and preserved in 70% ethanol. Muscles were then blotted dry and weighed using a precision balance (METTLER AE 100; ± 0.1 μg). Once the mass of each muscle (MM) was recorded, muscles were transferred to a petri dish and submerged in a 30% aqueous nitric acid solution for 24–48 h to dissolve the connective tissue surrounding the muscular fibres (Loeb & Gans, 1986). Next fibres were teased apart and the nitric acid was replaced by a 50% aqueous glycerol solution. Each muscle was observed with a binocular loupe (Leica WILD M3Z 308,700), illuminated by light arms (Intralux® 4000, Geprüfte Sicherheit) and the fibres were separated using forceps and photographed. Fibre lengths (mm) were measured using ImageJ (Abràmoff et al., 2004).

### 2.3 Bite forces and biomechanical model

**FIGURE 1:**
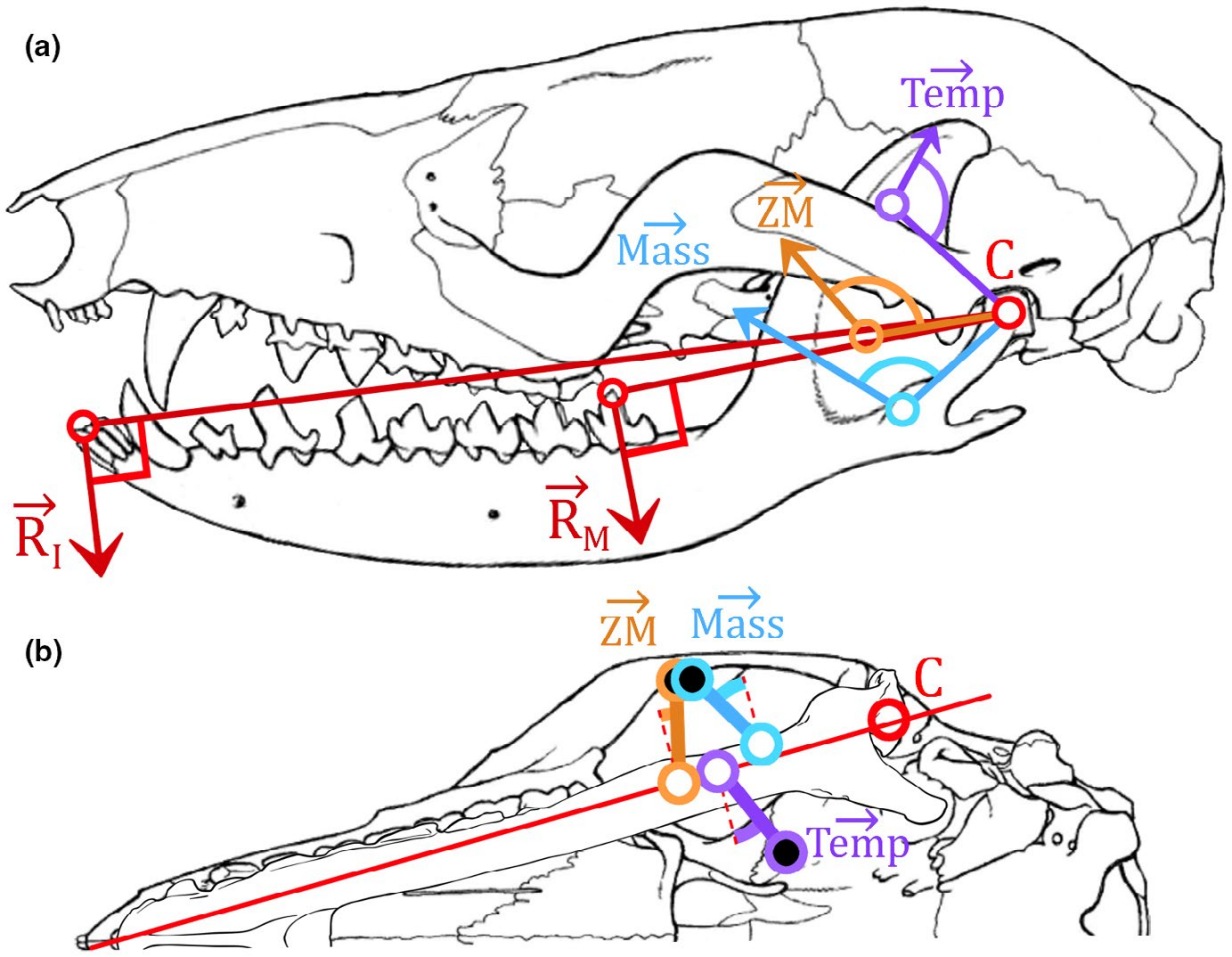
Biomechanical bite model applied on *Marmosa murina* relying on the static equilibrium of the masticatory muscular forces in lateral (a) and ventral (b) view. In lateral view, the mandible has been placed at the optimal gape. Skull and mandible modified from Voss and Jansa (2009), and mandible in the ventral view (b) drawn by Lola Lainé. The black and the white dots, respectively, represent the origin on the skull and the insertion on the mandible of the muscles. The angles shown are $\theta_{i}$ in (a) and $\varphi_{i}$ in (b). C, Condylar process; Mass, Masseter muscle; R_I_ and R_M_, Resultant force on the incisors (I) and the last molar (M); Temp, Temporal muscle; ZM, Zygomaticomandibular muscle.

The biomechanical model has been established on the R software (2021) and was inspired by those previously described, relying on the static equilibrium of the masticatory system (e.g., Cleuren et al., 1995; Herrel et al., 1998b) (Figure 1a,b). We first calculate the physiological cross‐sectional area PCSA. Although some authors use the RPCSA instead of the PCSA by “reducing” the PCSA by the pennation angles with the muscles, others argue against applying RPCSA to the masticatory system (Hartstone‐Rose et al., 2018). Next, we calculate the three‐dimensional maximum force vector $F_{3\text{Dmax}\left(i\right)}$ for each muscle i with the modified formula of Schumacher (1961) by using (i) the fibre length ${\mathrm{FL}}_{\left(i\right)}$, (ii) the muscle mass ${\mathrm{MM}}_{\left(i\right)}$, (iii) a muscle density $d_{M}$, (iv) a mass correction coefficient of 1.692 due to the average loss of 40.91% mass from formalin fixation and ethanol preservation for at least 6 months (Leonard *et al.*, 2022), (v) and the muscle stress FMS, estimated at 30 N.cm^−2^ (Close, 1972):

(1) $$F_{3\text{Dmax}\left(i\right)}=\frac{\mathrm{FMS}*{\mathrm{MM}}_{\left(i\right)}*1.692}{{\mathrm{FL}}_{\left(i\right)}*d_{M}}.$$

The muscle density $d_{M}$ value of 1.0597 g.cm^−3^ or 1.0564 g.cm^−3^ was previously estimated by Mendez and Keys (1960) and Murphy and Beardsley (1974), respectively. The first authors used muscles from the lower limb of mature rabbits and dogs, and the second ones measured density of soleus muscles of adult cats while evaluating its mechanical properties. Leonard et al. (2021) pointed out the limitations and the unspecified methods in these references, and thus defined more specific values according to the anatomical region studied. By dissecting the muscles of the head, forelimb, hindlimb, and trunk of the New Zealand white rabbit *Oryctolagus cuniculus* Linnaeus, 1758, they estimate a general muscle and a head muscle density of 1.0558 g.cm^−3^ and 1.0518 g.cm^−3^, respectively. They also calculated the muscle density of the masseter muscle, 1.0582 g.cm^−3^, but we here use the average head muscle density for all our masticatory muscles, since we do not have muscle density values for all the other masticatory muscles.

We then estimate the two‐dimensional maximum force $F_{2\text{Dmax}\left(i\right)}$ for each muscle i, *i.e*. only the dorsoventral and anteroposterior components of the 3D vector that impact mandible rotation. We discount the mediolateral components of muscle force because they cancel each other out in bilateral biting, nor do they contribute to mandibular rotation, which is involved in jaw closure or opening. The projection of the muscle vector i in the (dorsoventral, anteroposterior) plane is made by (i) measuring the angle $\varphi_{i}$ between the line of action of muscle i and the medio‐lateral axis of the mandible (Figure 1b), and by (ii) subtracting the medial‐lateral component from $F_{3\text{Dmax}\left(i\right)}$:

(2) $$F_{2\text{Dmax}\left(i\right)}=\sqrt{{F_{3\text{Dmax}\left(i\right)}}^{2}-{\left(F_{3\text{Dmax}\left(i\right)}*\cos\left(\varphi_{i}\right)\right)}^{2}}.$$

Finally, with the establishment of the moment equilibrium equation, the resultant force $R_{i-\mathrm{BP}}$ at the bite point is calculated by using for each muscle i, (i) the lever arm distance, from the centroid of the muscular insertion area to the centre of condylar process, $D_{I-C\left(i\right)}$, (ii) the angle $\theta_{i}$ between $\vec{D_{I-C\left(i\right)}}$ and the line of action of muscle i (Figure 1a), and (iii) the distance from the centre of condylar process to the bite point (BP), *i.e.* the incisors or the last molar, $D_{C-\mathrm{BP}}$:

(3) $$R_{i-\mathrm{BP}}=\frac{D_{I-C\left(i\right)}*\sin\left(\theta_{i}\right)*F_{2\text{Dmax}\left(i\right)}}{D_{C-\mathrm{BP}}}.$$

The last step to estimate the maximum bite force at the incisors and the last molar, $F{\left(\text{calc}\right)}_{I}$ and $F{\left(\text{calc}\right)}_{M}$, respectively, is to double the sum of all calculated $R_{i}$ for each studied tooth, in order to simulate a tetanic contraction of all masticatory muscles on both sides on the skull:

(4) $$F{\left(\text{calc}\right)}_{\mathrm{BP}}=2*\sum R_{i-\mathrm{BP}}.$$

To establish the optimal gape, we then simulate the opening of the mouth by reapplying equations ((1), (2), (3))–(4) after rotating all points of interest located on the mandible at each 0.5 degrees step, *i.e.* the centroid of insertion area of all muscle and the bite points (incisors and the last molar). The centre of rotation is located at the centre of the condylar process. A new value of $F\left(\text{calc}\right)$ is calculated for each 0.5 degrees of rotation. The optimal gape is obtained when the value of $F\left(\text{calc}\right)$ reaches its peak, *i.e.* at the angle before which the recalculated value has decreased after one rotation. At the optimal gape angle, if the final calculated maximum bite force value $F\left(\text{calc}\right)$ is not significantly close to the maximum bite force value measured on living specimens $F\left(\text{alive}\right)$, we reapply the equations ((1), (2), (3))–(4) by increasing or decreasing the muscle stress FMS of 30 N.cm^−2^ (Close, 1972) until the two force values are similar.

### 2.4 Sensitivity of the model

To study the contribution of each parameter to the maximum bite force for *Marmosa murina*, we varied each parameter by ±5% for each muscle, and then observed by how much the value of the calculated maximum bite force $F\left(\text{calc}\right)$ is affected. The six main parameters of our biomechanical bite model are, for each muscle i: (i) the lever arm distance, from the centroid of the muscular insertion area to the centre of condylar process, $D_{I-C\left(i\right)}$; (ii) the angle $\theta_{i}$ between $\vec{D_{I-C\left(i\right)}}$ and the line of action of muscle i; (iii) the muscle mass ${\mathrm{MM}}_{\left(i\right)}$; (iv) the fibre length ${\mathrm{FL}}_{\left(i\right)}$; (v) the angle $\varphi_{i}$ between the line of action of muscle i and the mediolateral axis of the mandible; and (vi) the distance from the centre of condylar process to the bite point (BP), *i.e.* the incisors or the last molar, $D_{C-\mathrm{BP}}$.

## 3 RESULTS

### 3.1 Dissection and anatomy

**FIGURE 2:**
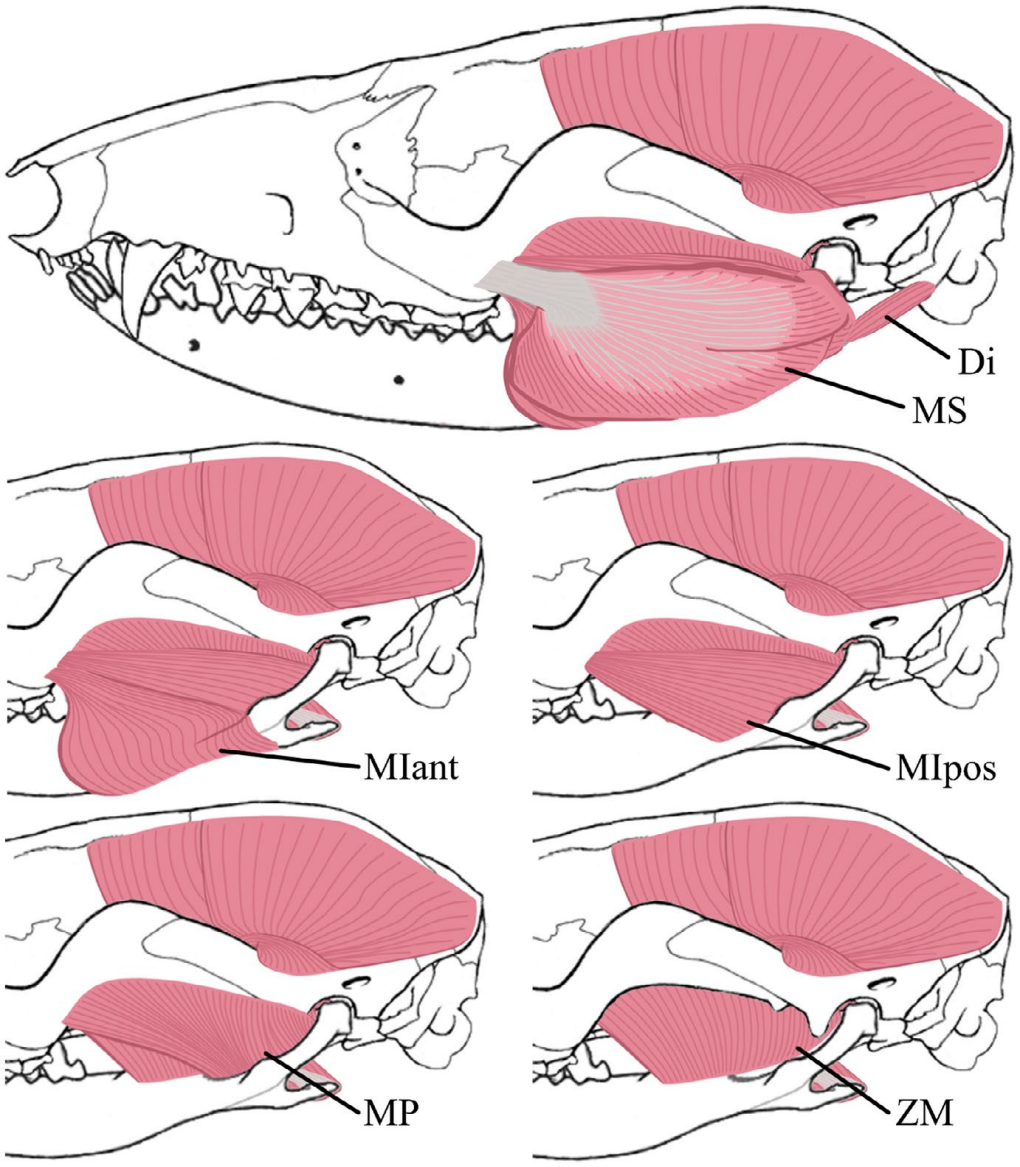
Superficial masticatory muscles identified in *Marmosa murina*, in lateral view. Skull and mandible modified from Voss and Jansa (2009). Di, Digastric; MIant, *Masseter intermediate anterior*; MIpos, *Masseter intermediate posterior*; MP, *Masseter profound*; MS, *Masseter superficial*; ZM, Zygomaticomandibularis.

**FIGURE 3:**
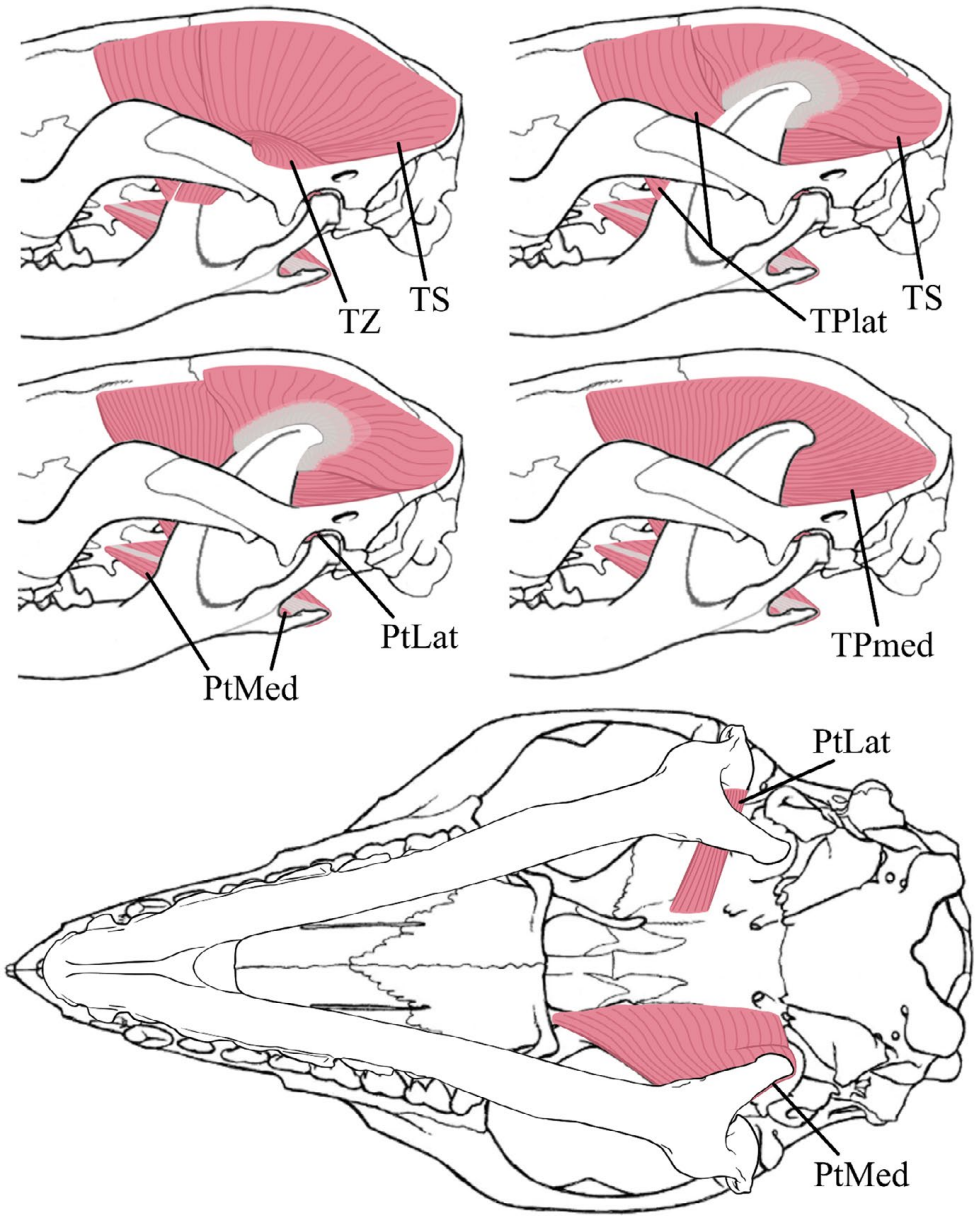
Deep masticatory muscles identified in *Marmosa murina*, in lateral and ventral view. The Digastric, Masseter, and Zygomaticomandibular muscles were completely removed to study the deep masticatory muscles. Skull and mandible modified from Voss and Jansa (2009), and mandible in the ventral view drawn by Lola Lainé. PtLat, Pterygoid lateral; PtMed, Pterygoid medial; TS, Temporal superficial; TPlat, Temporal profound lateral; TPmed, Temporal profound medial; TZ, Temporal zygomatic.

**FIGURE 4:**
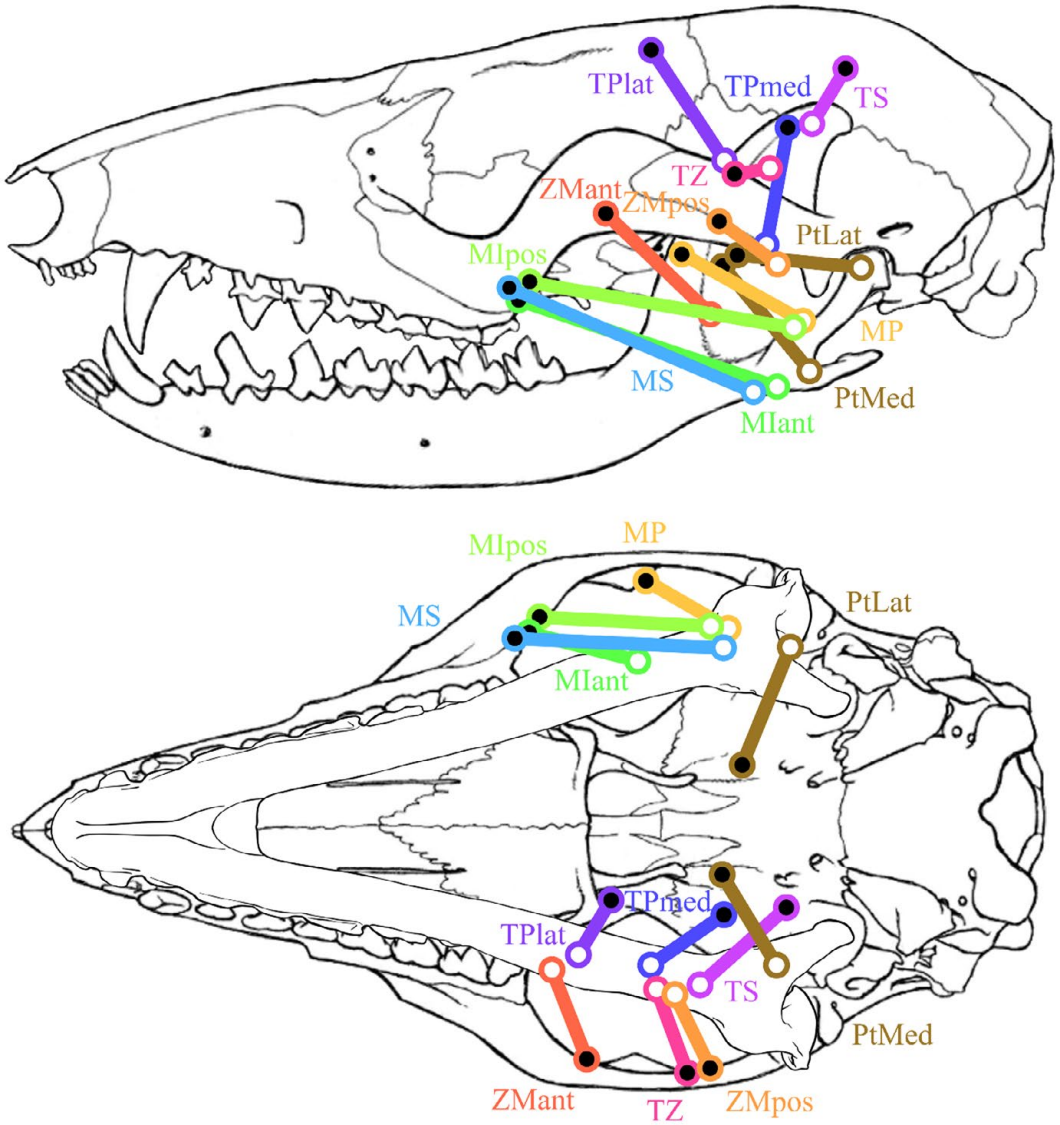
All lines of action, *i.e.* vectors from the skull origin to the mandibular insertion, of the masticatory muscles implied in the closing of the jaw, except for *M. pterygoideus lateralis*, and identified by dissection in *Marmosa murina*, in lateral and ventral view. In lateral view, the mandible has been placed at the optimal gape. Skull and mandible modified from Voss and Jansa (2009), and mandible in the ventral view drawn by Lola Lainé. MIant, Masseter intermediate anterior (lime); MIpos, Masseter intermediate posterior (light green); MP, Masseter profound (yellow); MS, Masseter superficial (azure); PtLat, Pterygoid lateral (brown); PtMed, Pterygoid medial (brown); TS, Temporal superficial (fuchsia); TPlat, Temporal profound lateral (purple); TPmed, Temporal profound medial (dark blue); TZ, Temporal zygomatic (pink); ZMant and ZMpos, Zygomaticomandibularis anterior (orange) and posterior (light orange).

**FIGURE 5:**
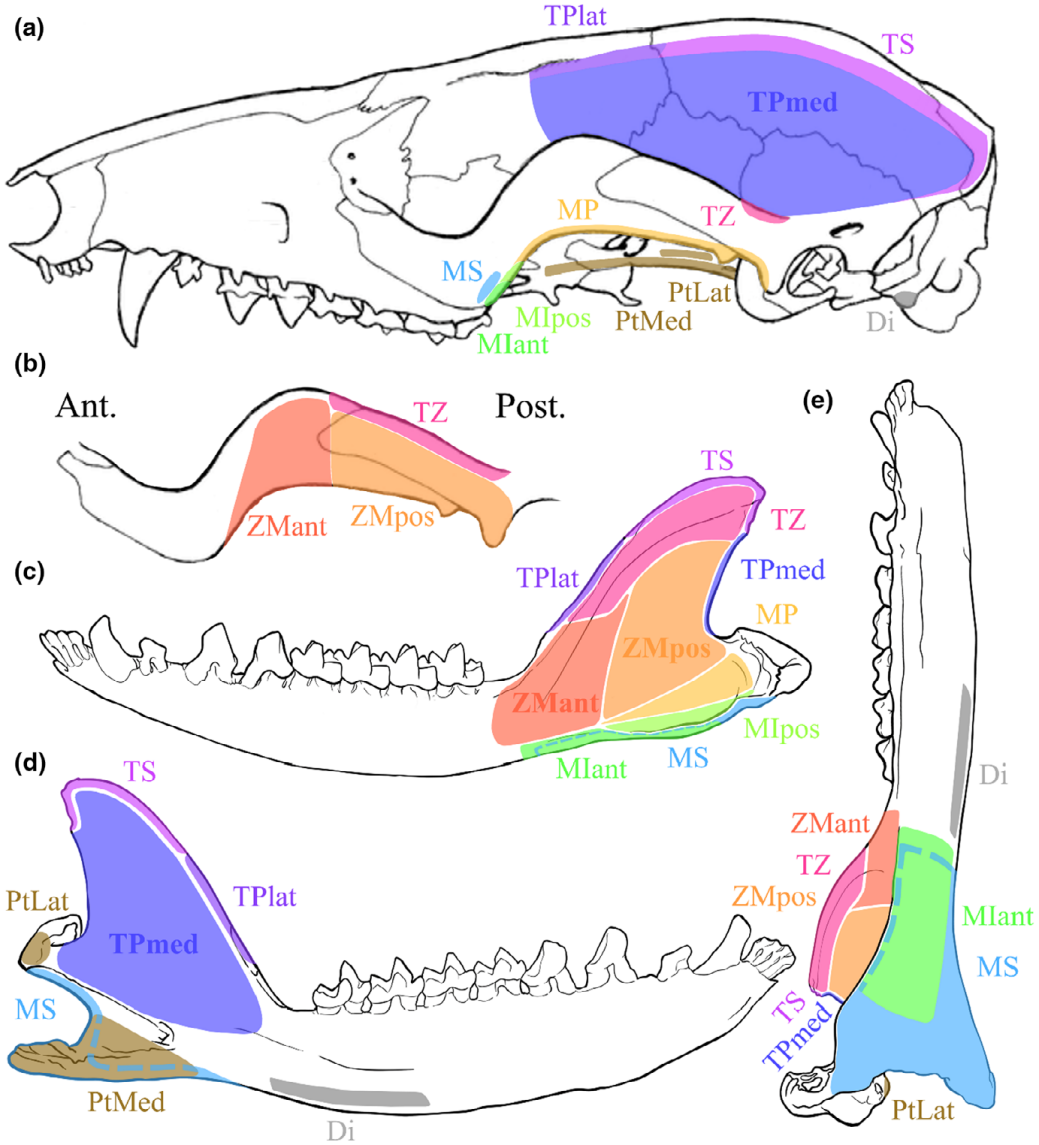
Skull origin and the mandibular insertion areas of the masticatory muscles of *Marmosa murina* identified by dissection. (a) Lateral view of the skull; (b) medial side of the zygomatic arch; (c) lateral side, (d) medial side and (e) ventral side of the mandible (a,b: Modified from Voss and Jansa (2009); (c–e) Mandible drawn by Lola Lainé). Di, Digastric (grey); MIant, Masseter intermediate anterior (lime); MIpos, Masseter intermediate posterior (light green); MP, Masseter profound (yellow); MS, Masseter superficial (azure); PtLat, Pterygoid lateral (brown); PtMed, Pterygoid medial (brown); TS, Temporal superficial (fuchsia); TPlat, Temporal profound lateral (purple); TPmed, Temporal profound medial (dark blue); TZ, Temporal zygomatic (pink); ZMant, Zygomaticomandibularis anterior (orange); ZMpos, Zygomaticomandibularis posterior (light orange).

The masticatory muscles were subdivided as follows: the masseter muscle, comprising the *M. masseter superficialis* (MS) and the *M. masseter profundus* (MP); the zygomaticomandibular muscle (ZMant and ZMpost, for anterior and posterior, respectively); the temporalis muscle, comprising the *M. temporalis superficialis* (TS), the *M. temporalis pars suprazygomatica* (TZ), and the *M. temporalis profundus* (TPlat and TPmed, for lateral and medial, respectively); the *M. pterygoideus*, comprising the *M. pterygoideus lateralis* (PtLat, also called *M. pterygoideus externus*) and the *M. pterygoideus medialis* (PtMed, also called *M. pterygoideus internus*); the jaw opener, the *M. digastricus* (Di) was also dissected. An intermediate part (*pars intermedius*) of the *M. masseter* was observed during dissection and detailed individually. The *M. masseter intermedius* is quite distinct and considered as a part of the masseter muscle, lying between *M. masseter superficialis* (MS) and *M. masseter profundus* (MP). The weights (MM), relative mass percentages among the masticatory system (%mast), the fibre lengths (FL), and the PCSA of each masticatory muscle for M1496 and M2851 are shown in Table 1. All identified muscles, from the most superficial to the deepest, are represented in Figures 2 and 3, as is their respective line of action in Figure 4 and their respective origin and insertion areas in Figure 5. All muscular descriptions below refer to our case study, results from the dissection of the *Marmosa murina* specimens mentioned.

**TABLE 1:** Masticatory muscle masses (g), relative masses (%), fibre lengths (mm), and PCSA values (cm^2^) of *Marmosa murina* specimens included in this study.

Masticatory muscles of *Marmosa murina*	M1496, adult, male				M2851, adult, male			
	Dry weights/muscle mass MM (g)	Relative mass percentages %mast	Fibre lengths FL (mm)	PCSA (cm^2^)	Dry weights/muscle mass MM (g)	Relative mass percentages %mast	Fibre lengths FL (mm)	PCSA (cm^2^)
*M. digastricus* Di	0.0205	—	6.689	0.0291	0.0166	—	4.673	0.0338
*M. masseter superficialis* MS	0.0513	17.40%	4.606	0.1059	0.0485	16.85%	3.138	0.1469
*M. masseter intermedius ant* MIant	0.0071	2.41%	4.272	0.0158	0.0072	2.50%	3.495	0.0196
*M. masseter intermedius post* MIpos	0.0166	5.63%	2.964	0.0532	0.0147	5.11%	3.500	0.0399
*M. masseter profundus* MP	0.0084	2.85%	3.046	0.0262	0.0091	3.16%	3.216	0.0269
*M. zygomaticomand. ant* ZMant	0.0232	7.87%	5.180	0.0426	0.0178	6.18%	4.411	0.0384
*M. zygomaticomand. pos* ZMpos	0.0088	2.99%	4.270	0.0196	0.0189	6.56%	3.607	0.0498
*M. temporalis pars suprazygomatica*. TZ	0.0065	2.20%	3.213	0.0192	0.0068	2.36%	2.453	0.0264
*M. temporalis superficialis* TS	0.0399	13.53%	5.472	0.0693	0.0483	16.78%	3.779	0.1215
*M. temporalis profundus lat* TPlat	0.0386	13.09%	5.369	0.0684	0.0342	11.88%	3.789	0.0858
*M. temporalis profundus med* TPmed	0.0708	24.02%	4.542	0.1482	0.056	19.45%	3.790	0.1405
*M. pterygoideus lat/ext* PtLat	0.0024	0.81%	2.043	0.0112	0.0028	0.97%	2.248	0.0118
*M. pterygoideus med/int* PtMed	0.0212	7.19%	2.109	0.0956	0.0236	8.20%	2.101	0.1068

#### 3.1.1 Muscle digastricus (Di)

The Di is a long but flat muscle (Figure 2), taking its origin at the exoccipital and at the mastoid process of the petrosal (Figure 5). It inserts on the medioventral border of the mandible from near the angular process to the P3 anteriorly (Figure 5). This parallel‐fibred muscle is divided into two parts, an anterior and a posterior one, connected by a strong tendon at the level of the medial edge of the *M. masseter superficialis*. Fibres are directed along the anteroposterior axis along the entire muscle. The anterior part of the *M. digastricus* covers the insertion of the *M. mylohyoideus* at the level of the mandible. The digastric muscle represents about 5.98% of the total muscle mass of the masticatory apparatus in our morphological sample (Table 5).

#### 3.1.2 *M. masseter superficialis* (MS)

The MS is a thick, round, and superficial muscle in the cheek region of *Marmosa murina* (Figure 2). It accounts for about 16.10% of the total muscle mass in our morphological sample (Table 5). The fibres converge anteriorly and originate by means of a strong tendon attached to the lateral posterior maxillary extremity, very close to the posterior end of the M4 (Figure 5). This tendon extends as far as the anteroposterior level of the M1/P3. This muscle covers the *M. masseter intermedius* and inserts on the ventral edge of the mandible (Figure 5). A part of it inserts ventrally on the aponeurosis covering the *M. masseter intermedius anterior*, and another part slightly on the medial edge of the angular process, laying on and covering part of the *M. pterygoideus medialis* (Figure 5).

#### 3.1.3 *M. masseter intermedius* (MI)

The MI accounts for about 7.36% of the total muscle mass in our morphological sample. Its anterior (MIant) and posterior (MIpos) parts represent about 2.31% and 5.05%, respectively (Table 5). The *M. masseter intermedius anterior* (MIant) is a triangularly shaped muscle (Figure 2) that inserts at the lateroventral edge of the angular process, more anteriorly than the MS (Figure 5). It does, however, not overlap the extremity of the angular process. Its fibres converge towards its origin at the posterior edge of the descending process of the jugal, near the jugal‐maxillary suture, medially next to the origin of the *M. masseter superficialis* (MS) (Figure 5). The *M. masseter intermedius posterior* (MIpos) is a thin crescent‐shaped muscle (Figure 2) that inserts inside and along the ventral line of the masseteric ridge (Figure 5). Its fibres are fan‐shaped and closely associated with the *M. masseter profundus* (MP), covering nearly its entire lateral surface. The restricted origin of the posterior intermediate masseter is on the ventral edge of the zygomatic arch, more dorsal and posterior than the insertion area of the anterior part of this muscle (Figure 5).

#### 3.1.4 *M. masseter profundus* (MP)

The deep MP muscle is roughly half the weight of the MIpos, with dorsoventrally oriented fibres. This flat, fan‐shaped muscle (Figure 2), accounts for about 2.82% of the total muscle mass in our morphological sample (Table 5). It originates along most of the ventral edge of the jugal and squamosal and is positioned just under the *M. masseter intermedius posterior* (MIpos) (Figure 5). It inserts at the ventral side of the masseteric ridge, from the condyloid process to the anteroventral border of the fossa masseterica (Figure 5). The distinction between the *M. masseter profundus* (MP) and the *M. masseter intermedius posterior* (MIpos) can be difficult to observe. The *M. masseter profundus* (MP) is positioned on top of an aponeurosis, separating the fibres of this muscle and those from the *M. zygomaticomandibularis* (ZM).

#### 3.1.5 *M. zygomaticomandibularis* (ZM)

The ZM is externally covered by a thin aponeurosis, allowing a clear distinction between this muscle and the MP. It is divided into an anterior part (ZMant) and a posterior part (ZMpos), accounting for around 6.62% and 4.47% of the total muscle mass in our morphological sample, respectively (Table 5). The ZM is more dorsoventrally oriented than the masseter muscle (Figure 2). The *M*. *zygomaticomandibularis posterior* (ZMpos) is a parallel‐fibred muscle that is much thicker than the deep (MP) and posterior intermediate (MIpos) masseter parts. It originates on the posterior half of the mesial side of the zygomatic arch, beneath the origin of the *M. temporalis pars suprazygomatica* (TZ) (Figure 5). It inserts within the posterior part of the fossa masseterica, lying under the *M. masseter profundus* (MP) (Figure 5). The *M. zygomaticomandibularis anterior* (ZMant) is very similar to the ZMpos but its ventral part is less thick. This muscle inserts into the anterior part of the fossa masseterica, from the insertion area of the ZMpos to on the dorsal border of the masseteric ridge (Figure 5). Its parallel fibres originate on the anterior half of the mesial side of the zygomatic arch (Figure 5).

#### 3.1.6 *M. temporalis pars suprazygomatica* (TZ)

The TZ accounts for about 2.15% of the total muscle mass in our morphological sample (Table 5). It originates along the dorsal extremity of the dorsal and mesial edge of the zygomatic process of the squamosal, positioned partly on the lateral side of the zygomatic arch (Figure 5). The muscle extends to a partial connection with the TS, close to the posterior border of the coronoid process of the mandible. It is attached on most of the dorsal half of the masseteric ridge (Figure 5). Its fibres extend from the masseteric ridge to the dorsoposterior end of the mesial side of the zygomatic process of the squamosal (Figure 3).

#### 3.1.7 *M. temporalis superficialis* (TS)

The TS is one of the most strongly developed muscles of the masticatory apparatus of *M. murina*, accounting for about 14.23% of the total muscle mass in our morphological sample (Table 5). In this species, we find that this flat fan‐shaped muscle (Figure 3) originates just above the *M. temporalis profundus medialis* (TPmed) along the temporal line near the sagittal crest (Figure 5). It ranges from the interparietal to the parietal, and then anteriorly extends to near the frontoparietal suture (Figure 5). Its fibres converge towards the insertion on the dorsal edge of the coronoid process (Figure 5). The TS is composed of two layers of fibres, and the deepest is connected to a wide aponeurosis, named *planum tendineum temporalis* by Turnbull (1970), on which all its medial‐most fibres converge (Figure 3). These fibres extend over the TPmed. The superficial temporal muscle is covered by a thin aponeurosis, distinguishing the temporalis from the zygomaticomandibularis muscles.

#### 3.1.8 M. temporalis profundus (TP)

The TP accounts for about 32.21% of the total muscle mass in our morphological sample (Table 5). The lateral (TPlat) and medial (TPmed) parts represent about 11.75% and 20.46%, respectively, of the total muscle mass (Table 5). It is the largest muscle of the masticatory apparatus of *M. murina* (Figure 3). The origin of the *M. temporalis profundus laterali*s (TPlat) starts from near the fronto‐parietal suture to the postorbital process of the frontal (Figure 5). The mostly‐parallel fibres of this thin muscle converge along the anterodorsal edge of the coronoid process, just anterior to the aponeurosis of the TS (Figure 5). The *M. temporalis profundus medialis* TPmed originates slightly below the origin of the TS and the *M. temporalis profundus laterali*s (TPlat) but does not extend more anteriorly (Figure 5). This muscle originates on an area that extends across the parietal, frontal, squamosal, and alisphenoid (Figure 5). Its parallel fibres run from the origin to the medial side of the coronoid process (Figure 3), inserting on most of the coronoid process, from the insertion area of the TS to the anteroventral end of the mandibular ramus, and posteriorly on the coronoid and as far as near the condyloid process (Figure 5). This very thick muscle has the largest insertion area in the masticatory apparatus in *M. murina*.

#### 3.1.9 *M. pterygoideus medialis* (PtMed) and *M. pterygoideus lateralis* (PtLat)

The PtMed and the PtLat are two different parallel‐fibre muscles (Figure 3), accounting for 7.23% and 0.84% of the total muscle mass, respectively, in our morphological sample (Table 5). The first one is dorsally covered by a thin aponeurosis and is positioned ventral to the second one (Figure 3). It inserts on the medial inner side of the angular process (Figure 5). Its origin is located on the lateral border of the presphenoid and the pterygoid, close to the alisphenoid (Figure 5). It is attached more widely than the *M. pterygoideus lateralis* (PtLat), which originates on the lateral border of the alisphenoid (Figure 5). The lateral pterygoid is much thinner than the *M. pterygoideus medialis* (PtMed) (Figure 3), and its fibres insert on the medial side of the condyloid process (Figure 5).

### 3.2 Bite forces and biomechanical model

Table 2 summarizes the data calculated in our case study by the biomechanical model at a closed mouth, *i.e.* for each individualized muscle i, (i) the three‐dimensional maximum force $F_{3D\max\left(i\right)}$, (ii) the angle $\varphi_{i}$ between the line of action of the muscle and the medio‐lateral axis of the mandible, (iii) the two‐dimensional maximum force $F_{2D\max\left(i\right)}$, (iv) the angle $\theta_{i}$ between $\vec{D_{I-C\left(i\right)}}$ and the line of action of the muscle, and (v) the calculated resultant force $R_{i}$ on the incisors or the last molar. The MS, MIant, MIpos, TZ, TPmed, and PtMed retain most of their muscle strength after subtraction of their mediolateral component, because of their overall major anteroposterior or dorsoventral orientation. However, the TPlat and ZMant muscles lose most of their strength due to their strong mediolateral orientation (Figure 4).

**TABLE 2:** Summary of the data calculated by the biomechanical model with a mass correction coefficient of 1.692 (Leonard et al., 2022) and at closed mouth, *i.e.* with a gape angle of 0° and a muscle stress of 30 N.cm^−2^ (Close, 1972); For each muscle i, the three‐dimensional maximum force $F_{3\text{Dmax}\left(i\right)}$; the two‐dimensional maximum force vector $F_{2\text{Dmax}\left(i\right)}$; the angle $\varphi_{i}$ between the line of action of muscle i and the mediolateral axis of the mandible; the angle $\theta_{i}$ between the lever arm distance from the centroid of the muscular insertion area to the centre of condylar process $\vec{D_{I-C\left(i\right)}}$ and the line of action of muscle i; and the resultant force $R_{i-\mathrm{BP}}$ on the incisors (I) and the last molar (m4).

Masticatory muscles i of M2851	$F_{3\text{Dmax}\left(i\right)}$ (N)	Angle $\varphi_{i}$ (°)	$F_{2\text{Dmax}\left(i\right)}$ (N)	Angle $\theta_{i}$ (°)	$R_{i-I}$ (N)	$R_{i-M4}$ (N)
*M. masseter superficialis* MS	7.458	59.70	6.439	63.89	1.284	3.002
*M. masseter interm. ant* MIant	0.994	58.01	0.843	62.85	0.180	0.422
*M. masseter interm. post* MIpos	2.027	74.39	1.952	62.67	0.263	0.614
*M. masseter profundus* MP	1.366	37.12	0.824	89.60	0.121	0.282
*M. zygomaticomand. ant* ZMant	1.948	14.77	0.497	116.16	0.122	0.286
*M. zygomaticomand. pos* ZMpos	2.529	46.58	1.837	119.86	0.270	0.630
*M. temporalis pars suprazyg*. TZ	1.338	65.73	1.220	135.56	0.191	0.447
*M. temporalis superficialis* TS	6.168	37.93	3.792	94.56	0.992	2.319
*M. temporalis prof. lat* TPlat	4.356	8.14	0.617	68.43	0.151	0.352
*M. temporalis prof. med* TPmed	7.131	67.82	6.603	111.36	1.182	2.763
*M. pterygoideus lat/ext* PtLat	0.601	49.30	0.456	5.91	0.001	0.002
*M. pterygoideus med/int* PtMed	5.421	59.28	4.660	112.11	0.589	1.376

All the moments of the studied muscles are negative, favoring mouth closure in our model, but not the PtLat. Its positive value in our model shows its contribution to jaw opening. The role of this muscle as mandibular abductor has already been described in the mammalian masticatory musculature, for example, in dogs (Tomo et al., 1993) and primates (Hylander et al., 1987; Wall, 1999), but not yet specifically in marsupials. Furthermore, it has minuscule impact on the resultant force $R_{i-\mathrm{BP}}$ on the incisors (I) and the last molar (m4) (Table 2). Despite its potential link with the feeding ecology of a species through the functionally constrain maximum attainable gape (Dickinson, Pastor, *et al.*, 2021), we decide to no longer consider the PtLat in the calculation of the maximum bite force $F\left(\text{calc}\right)$.

**TABLE 3:** Comparison between the calculated maximum bite force value $F\left(\text{calc}\right)$ and the maximum bite force value measured on living specimens $F\left(\text{alive}\right)$ at different jaw configurations.

Bite point	*Marmosa murina* specimen	$F\left(\text{alive}\right)$ (N)	$F\left(\text{calc}\right)$ (N)			Value of optimal mouth gape (°)	Value of fitted muscle stress (N.cm^−2^)
			Closed mouth	At optimal mouth gape	At optimal mouth gape and FMS fitted		
Incisors (I)	M2851	13.467	10.688	10.754	13.467	6.5	37.567
	M1496		8.759	8.800	13.467	5.5	45.908
Last molar (m4)	M2851	37.185	24.992	25.148	37.185	6.5	44.360
	M1496		20.481	20.579	37.185	5.5	54.209

When comparing the maximum calculated bite force $F\left(\text{calc}\right)$ to the maximum in vivo bite force $F\left(\text{alive}\right)$ the in vivo forces are significantly higher. Table 3 presents these values for specimens M2851 and M1496 for the incisors (I) and the last molar (m4) at specific jaw configurations, *i.e.* (i) closed mouth, (ii) at optimal gape, and (iii) at the optimal gape with the fitted muscle stress. The value of optimal gape is estimated to be around 5.5° to 6.5° (represented in Figures 1 and 4). The value of the required muscle stress to converge the calculated forces on the in vivo forces varied from 37.567 to 54.209 N.cm^−2^, depending on the considered specimen and bite point.

### 3.3 Sensitivity of the model

The contribution of the six main parameters of our biomechanical bite model are summarized in Table 4. The three masticatory muscles most strongly contributing to the overall bite force are the MS, the TS, and the TPmed.

**TABLE 4:** Contribution (in %) of the six main parameter to the maximum bite force $F\left(\text{calc}\right)$ for *Marmosa murina* M2851. For each variation of these parameters, the three most impactful values are highlighted in bold. For example, varying only the muscle mass (MM) of the *Masseter superficialis* (MS) by −5% in the model will decrease the maximum bite force by 2.54%.

Model parameters	$D_{I-C\left(i\right)}$		Angle $\theta_{i}$		${\mathrm{MM}}_{\left(i\right)}$		${\mathrm{FL}}_{\left(i\right)}$		Angle $\varphi_{i}$		$D_{C-\mathrm{BP}}$	
	−5%	+5%	−5%	+5%	−5%	+5%	−5%	+5%	−5%	+5%	‐5%	+5%
MS	**−2.54**	**2.54**	**−3.06**	**1.81**	**−2.54**	**2.54**	**2.68**	**−2.42**	**−2.49**	**2.17**	**2.68**	**−2.42**
MIant	−0.36	0.36	−0.45	0.27	−0.36	0.36	0.38	−0.34	−0.37	0.33	0.38	−0.34
MIpos	−0.52	0.52	−0.69	0.43	−0.52	0.52	0.55	−0.50	−0.26	0.20	0.55	−0.50
MP	−0.22	0.22	0.02	−0.13	−0.22	0.22	0.23	−0.21	−0.48	0.45	0.23	−0.21
ZMant	−0.22	0.22	0.32	−0.43	−0.22	0.22	0.23	−0.21	−1.34	1.31	0.23	−0.21
ZMpos	−0.49	0.49	0.83	−1.07	−0.49	0.49	0.52	−0.47	−0.76	0.70	0.52	−0.47
TZ	−0.36	0.36	1.04	−1.21	−0.36	0.36	0.38	−0.34	−0.28	0.23	0.38	−0.34
TS	**−1.82**	**1.82**	0.62	−1.52	**−1.82**	**1.82**	**1.91**	**−1.73**	**−3.77**	**3.55**	**1.91**	**−1.73**
TPlat	−0.29	0.29	−0.28	0.13	−0.29	0.29	0.31	−0.28	**−3.24**	**3.20**	0.31	−0.28
TPmed	**−2.11**	**2.11**	**2.76**	**−3.80**	**−2.11**	**2.11**	**2.23**	**−2.01**	−1.48	1.22	**2.23**	**−2.01**
PtMed	−1.05	1.05	**1.41**	**−1.92**	−1.05	1.05	1.11	−1.00	−1.05	0.92	1.11	−1.00

## 4 DISCUSSION

### 4.1 Anatomy

In *Didelphis marsupialis* Linnaeus, 1758, the distinction between a superficial masseter and a deep masseter was observed as in *Dasyurus viverrinus* (Shaw, 1800), *Myrmecobius fasciatus* Waterhouse, 1836, and *Lasiorhinus latifrons* (Owen, 1845) (Crompton *et al.*, 2008; Thomas *et al.*, 2024). However, for the latter species the superficial part of the masseter was subdivided into two layers (Table 5). All other anatomical descriptions incorporated the MI into the MS, except Turnbull (1970) who separated the MIant and MIpos and included them in the MS and MP, respectively (Table 5). Aside from *Didelphis albiventris* Lund, 1840, *L. latifrons*, and *Phascolarctos cinereus* (Goldfuss, 1817), the *M. zygomaticomandibularis* was named and separated from the MP, but not divided into ZMant and ZMpos (Abreu & Astúa, 2024; Crompton *et al.*, 2008; Davison & Young, 1990; Thomas *et al.*, 2024; Turnbull, 1970).

**TABLE 5:** Differences in muscle nomenclature, percentages in total (%tot) and in the masticatory system, *i.e.* without the geniohyoid and the digastric (%MasSys) within the studied specimens. For *M. murina*, the %tot is the mean value of the M2851 and M1496 specimens.

	*Marmosa murina* Mean of M1496 and M2851			*Didelphis marsupialis* (Turnbull, 1970)			*Didelphis albiventris* (Abreu & Astúa, 2024)			*Dasyurus viverrinus* (Thomas *et al.*, 2024)		
Muscles	Abrev.	%mean	%MasSys	Term.	%	%MasSys	Term.	%	%MasSys	Term.	%	%MasSys
Digastricus	Di	5.98%	—	Digastricus	4.70%	—	Digastricus	3.11%	—	Digastricus	6.21%	—
Masseter superficialis	MS	16.10%	17.13%	Superficial masseter	14.30%	14.99%	Masseter pars superficialis	20.98%	21.65%	Mass. pars superficialis (sup. mass)	16.50%	17.59%
Masseter intermediaris	MIant	2.31%	2.45%									
	MIpos	5.05%	5.37%	Deep masseter	9.30%	9.75%				Mass. pars profunda (int. mass)	7.69%	8.20%
Masseter profundus	MP	2.82%	3.00%				Masseter pars profunda	10.01%	10.33%			
Zygomatico‐mandibularis	ZMant	6.62%	7.04%	Zygomatico‐mandibularis	9.00%	9.43%				Zygomatico‐mandibularis (deep mass)	14.80%	15.78%
	ZMpos	4.47%	4.75%									
Temporalis pars supra‐zygomatica	TZ	2.15%	2.28%	Temporalis pars zygomatica	19.70%	20.65%	Temporalis pars superficialis	39.87%	41.15%	Temp. pars supra‐zygomatica	1.00%	1.07%
Temporalis superficialis	TS	14.23%	15.14%	Temporalis						Temp. pars superficialis	11.50%	12.26%
Temporalis profundus (lateralis & medialis)	TPlat	11.75%	12.49%	Deep portion of the temporal	34.50%	36.16%	Temporalis pars profunda	21.07%	21.75%	Temp. pars intermedius	17.90%	19.09%
	TPmed	20.46%	21.76%							Temp. pars profunda (deep temp.)	16.82%	17.94%
Pterygoideus lateralis /externus	PtLat/ext	0.84%	0.89%	Pterygoid externus	1.60%	1.68%	Not mentionned	—	—	Pterygoideus lateralis (lat. pt.)	1.18%	1.26%
Pterygoideus medialis/internus	PtMed/int	7.23%	7.69%	Pterygoid internus	7.00%	7.34%	Pterygoideus medialis	4.96%	5.12%	Pterygoideus medialis (med. pt.)	6.39%	6.81%

	*Marmosa murina* Mean of M1496 and M2851			*Myrmecobius fasciatus* (Thomas et al., 2024)			*Phascolarctos cinereus* (Davison & Young, 1990)			*Lasiorhinus latifrons* (Crompton et al., 2008)		
Muscles	Abrev.	%mean	%MasSys	Term.	%	%MasSys	Term.	%	%MasSys	Term.	%	%MasSys
Digastricus	Di	5.98%	—	Digastricus	11.19%	—	Digastric	7.60%	—	—	—	—
Masseter superficialis	MS	16.10%	17.13%	Mass. pars superficialis (sup. mass)	18.00%	20.51%	Superficial masseter	23.93%	26.69%	Superficial masseter external part	19.03%	19.02%
Masseter intermediaris	MIant	2.31%	2.45%									
	MIpos	5.05%	5.37%							Superficial masseter internal part	25.30%	25.29%
Massater profundus	MP	2.82%	3.00%	Mass. pars profunda (int. mass)	10.50%	11.96%	Deep masseter	18.86%	21.04%	Deep masseter	7.50%	7.50%
Zygomatico‐mandibularis	ZMant	6.62%	7.04%	Zygomatico‐mandibularis (deep mass)	10.00%	11.39%				Not mentionned	—	—
	ZMpos	4.47%	4.75%									
Temporalis pars supra‐zygomatica	TZ	2.15%	2.28%	Temporalis pars suprazygomatica	2.00%	2.28%	Temporalis superior	26.30%	29.33%	Temporalis: anterior (a tem) and posterior (p tem) (not superficial and profunda)	31.30%	31.29%
Temporalis superficialis	TS	14.23%	15.14%	Temp. pars superficialis	8.00%	9.11%						
Temporalis profundus (lateralis and medialis)	TPlat	11.75%	12.49%				Temporalis inferior	8.80%	9.81%			
	TPmed	20.46%	21.76%	Temp. pars profunda (deep temp.)	28.00%	31.90%						
Pterygoideus lateralis/externus	PtLat/ext	0.84%	0.89%	Pterygoideus lateralis (lat. pt.)	2.48%	2.83%	Lateral pterygoid	7.10%	7.92%	Lateral pterygoid: l pt	5.00%	5.00%
Pterygoideus medialis/internus	PtMed/int	7.23%	7.69%	Pterygoideus medialis (med. pt.)	8.79%	10.01%	Medial pterygoid	4.67%	5.21%	Med pt.: sup. (s mpt) and deep (d mpt)	11.90%	11.90%

The temporalis muscle in *M. murina* and *D. marsupialis* is composed of three layers: the suprazygomatic part, the superficial part, and the deep part (Figure I, respectively, TZ, TS, and TP). The entire muscle were described and divided by only two layers using different terminology in other marsupials: a “*M. temporalis pars superficialis*” and a “*M. temporalis pars profunda*” were recognised in *D. albiventris*, a “*M. temporalis superior*” and “*M. temporalis inferior*” for *P. cinereus*, and a “*M. temporalis anterior*” and “*M. temporalis posterior*” for *L. latifrons* (Davison & Young, 1990; Crompton et al., 2008; Abreu & Astúa, 2024; Table 5). However, Thomas et al. (2024) interpreted the temporalis muscle as being composed of four layers in *D. viverrinus*, but not in *M. fasciatus*: the “*M. temporalis pars suprazygomatica*,” the “*M. temporalis pars superficialis*,” the “*M. temporalis pars intermedius*,”, and the “*M. temporalis pars profunda*” (Table 5). The differences observed in the muscle layer terminology could indicate differences in the dissection and identification of the muscle bundles for each species, leading to comparisons of different physiological units, and therefore to inaccurate hypotheses.

No major differences were observed for the Ptlat and the PtMed between the different species described in the literature and our observations with the exception of differences in the percentages of total masticatory muscle mass (Table 5). This variability among these species may be related to phylogenetic position of the different species marsupials. Indeed, in terms of anatomical description and percentage of total masticatory muscle mass, the closest species to *M. murina* is *D. marsupialis*, both being Didelphidae.

However, the evolution of the traits measured and described in this paper could be influenced by a prominent role of sexual dimorphism in the *Marmosa murina* species, because we dissected two adult male specimens. A variable effect of a sexual dimorphism on the size and shape of the skull and mandible has been observed and estimated in the Didelphimorphia by Astúa (2010).

Moreover, differences in specimen preservation may impact the comparison of observed and dissected muscle tissues between different species. Leonard et al. (2022) highlighted the effect that the methods of specimen preservation can have on muscle volume loss. Among the studied specimens a 10% formalin fixation and a 70% ethanol preservation has been used for the specimens of *M. murina* and *Macropus giganteus* Shaw, 1790. Abreu and Astúa (2024) considered the mass correction identified by Leonard et al. (2022) by correcting the muscle mass of +40%. Thomas et al. (2024) also used a 10% formalin fixation, but added to 4% glycerol for 7 days, before transferring to 70% ethanol for storage. Yet, the specimen was first frozen before formalin fixation. Unlike our study, the specimen dissected by Tomo et al. (2007) had also been cut in half in the sagittal plane and its zygomatic arch completely removed. On the other hand, a dissection of freshly killed and embalmed specimens was carried out for *D. marsupialis*, and frozen, thawed, and refrozen specimens were used for *P. cinereus* (Davison & Young, 1990; Turnbull, 1970). Finally, no preservation method was detailed for *L. latifrons* (Crompton et al., 2008). The comparison of absolute masses of specimens with distinct preservation and dissection methods may lead to biases in the interpretation. In our case, the study of *Marmosa murina* masticatory muscles show muscular similarities with the other didelphids (Table 5).

### 4.2 Bite forces and biomechanical model

The optimal gape was calculated to be at around 6° for the two studied specimens (Table 3). Calculating the maximum bite force value at the incisors and the last molar, $F{\left(\text{calc}\right)}_{I}$ and $F{\left(\text{calc}\right)}_{M}$ for each specimen, leads to different values of the fitted muscle stress constant FMS (Table 3). A variation of 7.038 N for M2851 and 8.6 N for M1496 is observed between the application of the bite point on the incisors or on the last molar (Table 3). The muscle stress is lower in an incisors bite point model, in line with the static equilibrium of the masticatory system. These teeth being the most anterior in the dentition, it increases the distance between the centre of condylar process to the bite point (BP) $D_{C-\mathrm{BP}}$ and results in an overall decrease in the calculated maximum bite force value $F\left(\text{calc}\right)$ (Table 4). The use of anterior teeth has been associated with various behaviours in mammals, such as food selectivity and quantity in ungulates, killing technique in carnivores, and feeding rate in rodents (Freeman & Lemen, 2008; Ungar, 2010). Incisors are often small and less robust than molars, and applying excessive force on incisors could risk damaging them (Ungar, 2010). Molars, closer to the temporomandibular joint, allow more efficient force transmission from the jaw muscles and has evolved to withstand high loads (Greaves, 1982), making them ideal for generating and modelling maximum bite force. Furthermore, the last molar (m4) of Didelphidae and Dasyuromorphia is quantitatively considered the best analogue to the carnassial (m1) of Carnivora (Tarquini et al., 2020). Thus, in our model, more reliable results are obtained for the last molar model, showing a remaining difference in the fitted muscle stress FMS of 10.203 N between M1496 and M2851 (Table 3), surely due to their difference in body size.

Reevaluating the force muscle stress constant FMS for the case of marsupials has only been done once before, *i.e.* by Thomason et al. (1990). This author maximally stimulated the jaw adducting muscles of eight specimens of *Didelphis virginiana* Kerr, 1972 under anaesthesia and recorded the force generated between the first molars, estimating a mean stress of 317 kPa (or 31.7 N.cm^−2^) with a standard deviation of 81.9 kPa (or 8.19 N.cm^−2^). This value, compared with the range of 147–392 kPa (or 14.7 to 39.2 N.cm^−2^) obtained by Carlson and Walkie (1974) for other vertebrates, including mammals, may show that the muscle stress value estimated at 25 N.cm^−2^ (Nigg & Herzog, 1994) or 30 N.cm^−2^ (Close, 1972) for mammals may be close but distinct to that for marsupials. For comparison, the muscle stress for human masticatory muscles was estimated at 330 kPa (or 33 N.cm^−2^) by Weijs and Hillen (1985). In *Marmosa murina*, we calculated FMS values from two specimens, and obtained 44.360 N.cm^−2^ and 54.209 N.cm^−2^ (Table 3), which is much higher than the Thomason et al. (1990) values. These high values are rather surprising and suggest that our model underestimates true bite forces. As we used PCSA and not RPCSA, not taking pennation angle into account in our calculations could also lead to under‐ or overestimation of the muscle cross‐sectional areas. A close but different muscular density value, recently reestimated by Leonard et al. (2022) for mammals, could also be used specifically for marsupials in order to better understand their muscular adaptation and jaw evolution comparatively to those of placentals, and *in fine* build better‐fitting inferred models for extinct and fossil species. Alternatively, a higher muscle stress and muscle density different from other mammals could be an autapomorphy of *Marmosa murina*, or more broadly a synapomorphy of Didelphidae or marsupials, in line with the results for *Didelphis virginiana* (Thomason et al., 1990). Theses adaptations could possibly be related to their wide mouth opening (Paddle, 2000; Pemberton & Renouf, 1993; Attard et al., 2011).

### 4.3 Sensitivity of the model

Determining the contribution of the six main parameters of our biomechanical bite model (Table 4) allowed us to understand which parameter has the greatest influence on the calculated maximum bite force $F\left(\text{calc}\right)$. Two of them have an overall positive impact on the model: (i) the lever arm distance, from the centroid of the muscular insertion area to the centre of condylar process, $D_{I-C\left(i\right)}$ (Figure 1) and (ii) the muscle mass ${\mathrm{MM}}_{\left(i\right)}$. As these parameters increase, so does the resulting force applied at the bite point. On the other hand, two of the main parameters have an overall negative influence on the reconstruction of the maximum bite force: (i) the fibre length ${\mathrm{FL}}_{\left(i\right)}$ and (ii) the distance from the centre of condylar process to the bite point $D_{C-\mathrm{BP}}$ (Figure 1). As they are both placed as the denominators in the static equilibrium equation, an increase in either of these two parameters leads to a decrease in the resulting forces. Finally, the last two parameters, *i.e.* (i) the angle $\theta_{i}$ between $\vec{D_{I-C\left(i\right)}}$ and the line of action of muscle i and (ii) the angle $\varphi_{i}$ between the line of action of muscle i and the mandibular mediolateral axis, affect the model to a lesser degree. Depending of the angles values, the calculated maximum bite force $F\left(\text{calc}\right)$ will be affected positively or negatively, depending on the sign of the cosine and sine functions.

In most of the main parameter variations, the muscles having the greatest impact on the maximum bite force are the MS, the TS, and the TPmed (Table 4). These are the largest muscles within the masticatory apparatus of *Marmosa murina*, accounting for about 16.10%, 14.23% and 20.46% of the masticatory system, respectively (Table 5) and thus have a major impact on bite force generation as in other mammals (Brassard *et al.*, 2020, 2021, 2022; Dumont & Herrel, 2003; Herrel et al., 2008). Indeed, the muscles having the smallest impact on the maximum bite force, *i.e.* the *M. masseter profundus* and the *M. zygomaticomandibularis anterior* (Table 4), are among the thinnest muscles within the masticatory apparatus. However, the third least impacting muscle, *i.e.* the *M. temporalis profundus lateralis* (Table 4), is also the fourth largest (Table 5). It loses most of its strength due to its strong mediolateral orientation (Figure 4). The orientation of the muscle appears to be equivalent in importance to mass in the generation of bite force.

## 5 CONCLUSION

Our enhanced understanding of the masticatory musculature and the complex integrated system of the *Marmosa murina* jaw has enabled us to establish a 3D biomechanical model for the reconstruction of maximum bite forces, based on the principles of static equilibrium. With the exception of an intermediate layer in the masseter muscle (MI), the various muscles of the masticatory apparatus were clearly identified and individualised, with their areas of origin and insertion precisely recorded. The muscular disposition and proportions of *Marmosa murina* are closer to those of other didelphids than to those of other previously studied marsupials. The static equilibrium of the jaw and the optimal gap estimated around 6° support the use of the last molar (m4) to obtain maximum biting force, considered to be analogue to the carnassial tooth (m1) of Carnivora. However, PCSA was used instead of RPCSA in our model and an increase in muscle stress was required to match in vivo bite forces on the last molar (m4). Previous studies have determined constants standardised to all mammals, such as muscle stress and muscle density, based on analyses conducted only on some placental species. New constants may need to be determined specifically for marsupials to help for a better understanding of the jaw adaptation within Marsupialia.

## AUTHOR CONTRIBUTIONS

VD: Data acquisition, methods and data analysis, drafting of the manuscript, realisation of the figures and tables, critical revision of the manuscript, and approval of the article. AH: Data acquisition, help in the methods and data analysis, critical revision of the manuscript, and approval of the article. QG: Help in the methods and data analysis, approval of the article, and supervision. DG: critical revision of the manuscript, approval of the article, and supervision. ACF: Funding for data acquisition and approval of the article. SL: Study design, critical revision of the manuscript, approval of the article, funding and supervision.

## FUNDING INFORMATION

Vincent Decuypere and the Marsubite project, supervised by Sandrine Ladevèze, were supported by the Paris Ile‐de‐France Region—DIM “Matériaux anciens et patrimoniaux.” The 2017 field mission in French Guiana was funded by an ATM AGRIP grant of the MNHN to A‐C. Fabre.

## CONFLICT OF INTEREST STATEMENT

The authors declare no conflict of interest.

## Data Availability

The data that support the findings of this study are available from the corresponding author upon reasonable request.
